# ST-elevation Myocardial Infarction and Complete Heart Block in a Nitrate-free Patient using a New Emerging Substance called Rhino

**DOI:** 10.7759/cureus.7601

**Published:** 2020-04-09

**Authors:** Smit Deliwala, Tarek Haykal, Harsukh Dhillon, Saadia Shafi, Ghassan Bachuwa

**Affiliations:** 1 Internal Medicine, Hurley Medical Center, Michigan State University, College of Human Medicine, Flint, USA; 2 Internal Medicine, Hurley Medical Center, Michigan State University, Flint, USA

**Keywords:** stemi, rhino, pde5i, sildenafil, acs

## Abstract

The pervasive use of counterfeit sexual enhancement supplements is increasing worldwide. There are thousands of vendors on the internet while local gas stations and convenience stores are selling it across the United States (US). We report a case of right coronary artery ST-segment elevation and complete heart block in a nitrate-free patient shortly after consuming three 950 mg pills of a sexual enhancer known as rhino and completing sexual intercourse. Coronary angiography revealed 100% occlusion of the right coronary artery and a drug-eluting stent was inserted with a transvenous pacer that he tolerated well, and recovered without complications. The counterfeit drug has gained traction for its high user satisfaction and low cost among recreational customers. The Food and Drug Administration (FDA), through its MedWatch program, has frequently released citations to consumers warning them against rhino since 2015, while their labs have recognized two prime ingredients: sildenafil and tadalafil. Although adverse cardiac risk with this therapeutic class is low, we aim to parse out its temporal relationship with rhino, an enhancer containing 14-200 times the prescription limits of sildenafil and tadalafil.

## Introduction

Rhino was first reported as a sexual enhancer with tainted sildenafil and tadalafil properties by The Food and Drug Administration (FDA) by a series of public notifications through its medical product safety reporting program known as MedWatch program [1]. Rhino pills contain sildenafil and tadalafil at exceedingly high doses ranging between 14 - 200 times the recommended labeled dose to boost sales and increase customer satisfaction [2,3]. A United States (US) department of justice report looking into the origins of rhino revealed large shipments of sildenafil and tadalafil powder smuggled out of Hong Kong and China, followed by repackaging in the US using non-standardized methods tainted with contaminants [2,4]. Suppliers would often label these products as “no prescription necessary” as it was often sold to them under the guise of herbal supplements without disclosing its exact ingredients; it is widely found at local gas stations and convenience stores [3]. Sildenafil and tadalafil are phosphodiesterase-5 inhibitors (PDE5i) approved to treat erectile dysfunction (ED), one of the more common types of sexual dysfunction in males. PDE5i induces nitric-oxide mediated vasodilation through cyclic guanosine monophosphate (cGMP) to initiate and maintain an erection. PDE5i increases intracavernosal cGMP levels and as a result, increases both the number and duration of erections in men with ED. The most commonly reported adverse effects of sildenafil are headaches, dyspepsia, and facial flushing. The pooled data and postmarketing surveillance analysis after FDA approval of sildenafil revealed a finite risk of adverse cardiovascular outcomes, including acute myocardial infarction (MI) and sudden cardiac death, especially after completing sexual activity [5]. A significant limitation is its use in patients with known coronary artery disease (CAD) using nitrates, regardless of spatial frequencies. This case aims to provide a brief understanding of rhino and its temporal relationship to adverse cardiovascular risks in the setting of unregulated manufacturing methods, blending excessively high doses of PDE5i and contaminants, posing a substantial public health risk. Lastly, the contaminated market for sexual enhancement pills is proliferating, with recreational use growing in prevalence [2,4].

## Case presentation

**Figure 1:**
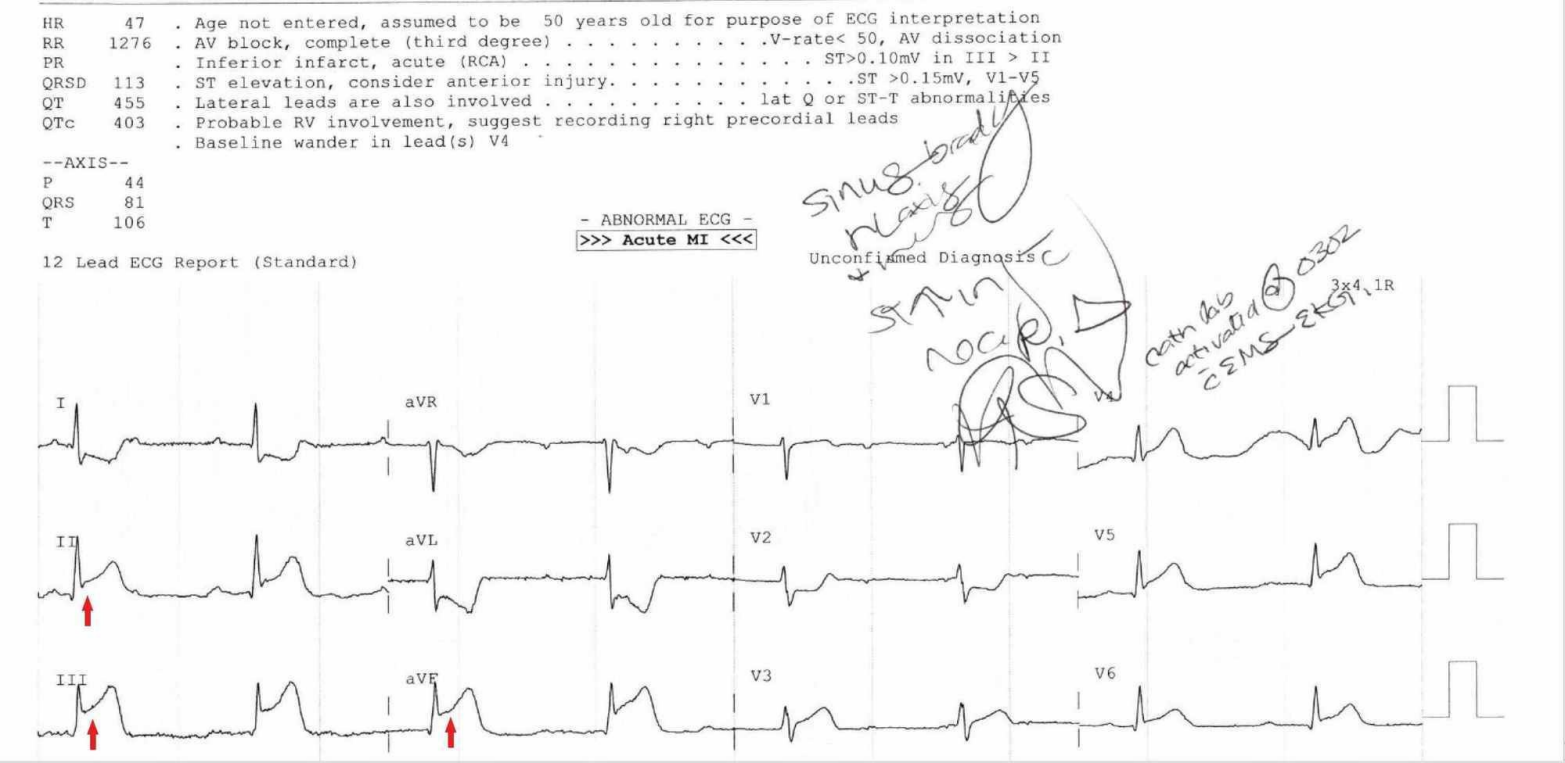
Electrocardiogram (ECG) by emergency medical services (EMS) revealing ST-elevation myocardial infarction (STEMI) and complete heart block (arrows) on scene

A 40-year-old male called the emergency medical services immediately after experiencing an unrelenting headache, nausea, and chest pain, that evolved shortly after consuming rhino and completing intercourse. He denied having a medical history, experiencing anginal symptoms in the past and denied using any medications, including herbal supplements. He denied a tobacco or alcohol history but endorsed to using alprazolam and various sexual enhancers for years. He stated that he began using sildenafil years prior despite not displaying any signs or symptoms of sexual dysfunction and using it solely for recreational purposes. Eventually, he switched to tadalafil when the effects began wearing off. On the insistence of his friends and looking for cost-effective methods, he switched to rhino in 2016, stating that the potency was producing satisfying results with lower costs compared to PDE5i. He would acquire rhino for $8 from multiple gas stations and endorsed being a chronic user of rhino, usually consuming one 950 mg pill before the intended use, but this particular time he took three pills. During his years of sexual enhancer use, he did endorse to frequently experiencing similar headaches in the past after use, although it was never to this degree, nor was it ever un-interrupting. A pre-hospital electrocardiograph (ECG) revealed an inferior ST-segment elevation myocardial infarction (STEMI) and complete heart block manifested by bradycardia (Figure 1).

**Figure 2:**
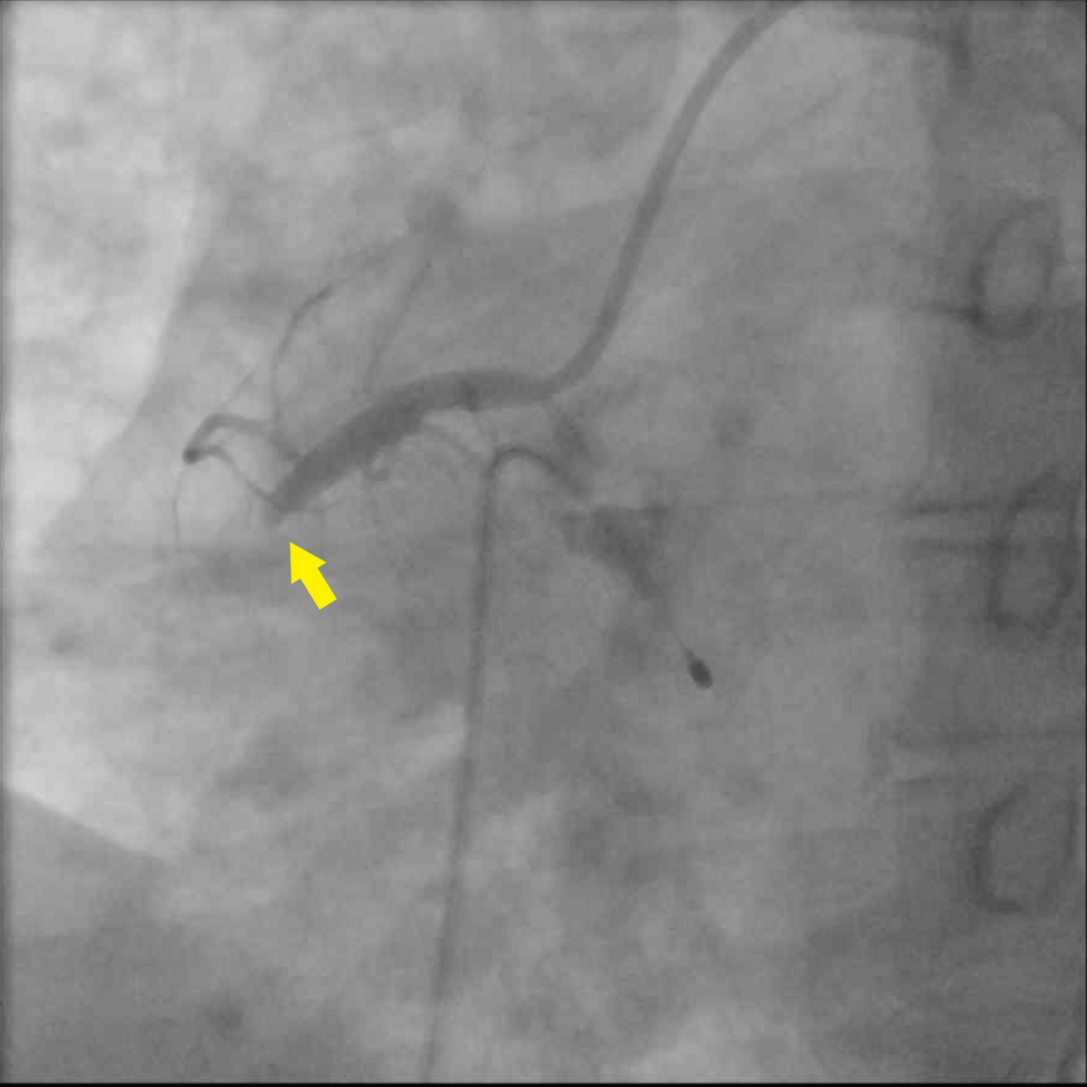
Cardiac catheterization demonstrating 100% stenosis of the right coronary artery and obstructed flow (arrow)

**Figure 3:**
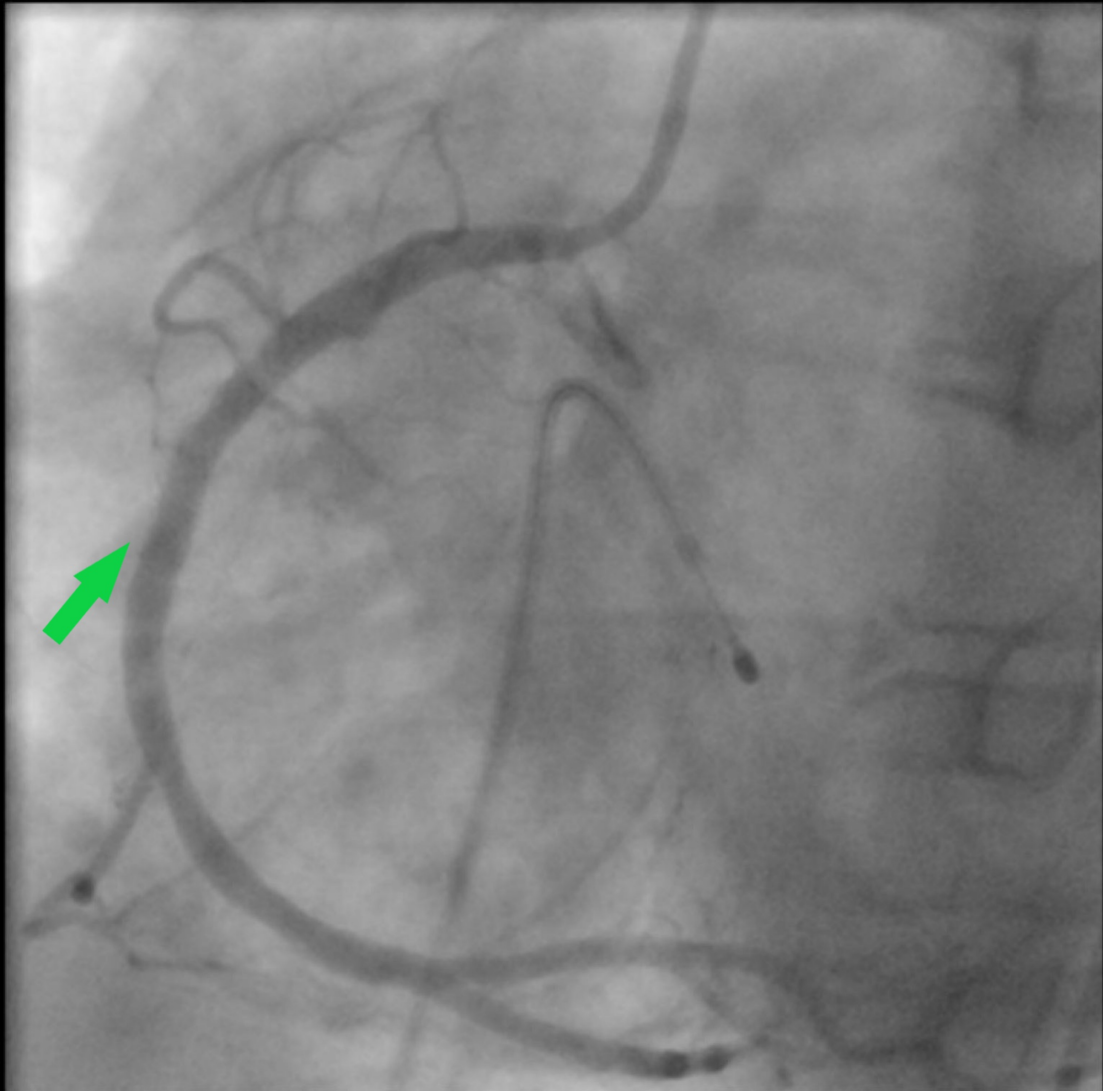
Cardiac catheterization post dilatation revealing patency of the right coronary artery (arrow)

**Figure 4:**
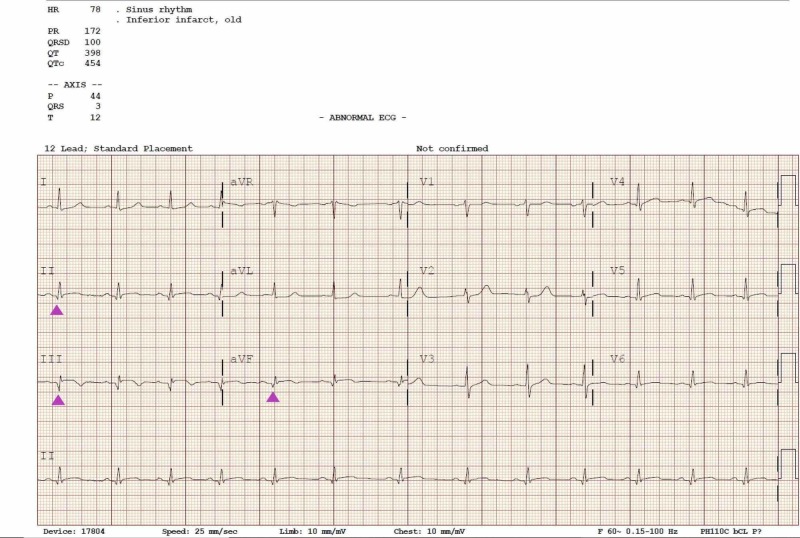
Electrocardiogram (ECG) post intervention demonstrating morphological changes and pathological q waves (arrowheads) consistent with a previous myocardial infarction (MI)

**Figure 5:**
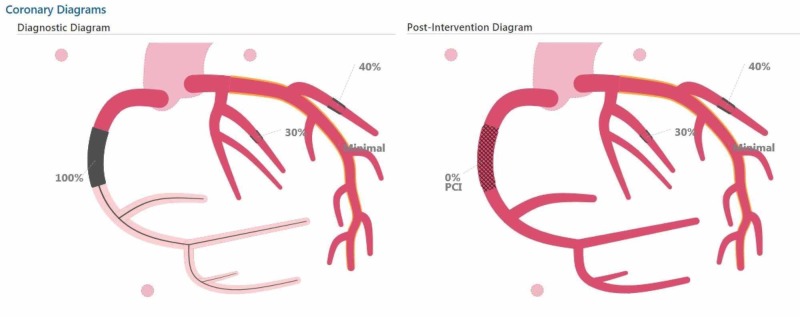
Reconstructed coronary diagram revealing anatomy before and after the intervention

His vitals while at the emergency department were blood pressure of 111/63 mm Hg, heart rate of 48 beats per minute, respiratory rate of 22, oxygen saturation of 99% on room air, and Glasgow Coma Scale of 15. On physical examination, he was diaphoretic with cold extremities and had precordial chest pain and brisk pulses throughout. A repeat ECG in the emergency department reported similar findings of inferior ST-segment elevations in leads II, III, aVF, V5, and V6 and ST-segment depressions in leads I, aVL, and V2 while high-sensitivity troponin I was 163.41 ng/mL. Notable labs was a white blood cell count of 14.3 K/UL without left shift, total cholesterol 184 mg/dL, triglycerides 283 mg/dL, high density lipoprotein (HDL) 26 mg/dL, low density lipoprotein (LDL) 101 mg/dL, mild transaminitis with aspartate aminotransferase at 307 U/L, and alanine aminotransferase at 117 U/L. Urine toxicology screen came back positive for benzodiazepines. As per protocol, the cardiac catheterization lab was activated, and the patient received fentanyl 50 mcg and a loading dose of aspirin 325 mg before the procedure. Coronary angiography revealed 100% occlusion with thrombolysis in myocardial infarction (TIMI) grade flow of 0 (Figure 2). He underwent percutaneous coronary intervention (PCI) with a drug-eluting stent (DES). Post dilation demonstrated 0% stenosis with a TIMI grade flow of 3 (Figure 3). He was simultaneously transvenously paced after demonstrating complete heart block morphology. After the procedure, he was started on dual antiplatelet therapy with aspirin 81 mg and ticagrelor 90 mg twice daily and was given analgesia. His sheath was removed shortly after and he recovered without any complication. An ECG completed post procedure revealed disappearance of ST changes and the presence of a Q wave consistent with a previous MI (Figure 4). A reconstructed diagram of the coronaries can be seen below (Figure 5). 

## Discussion

**Figure 6:**
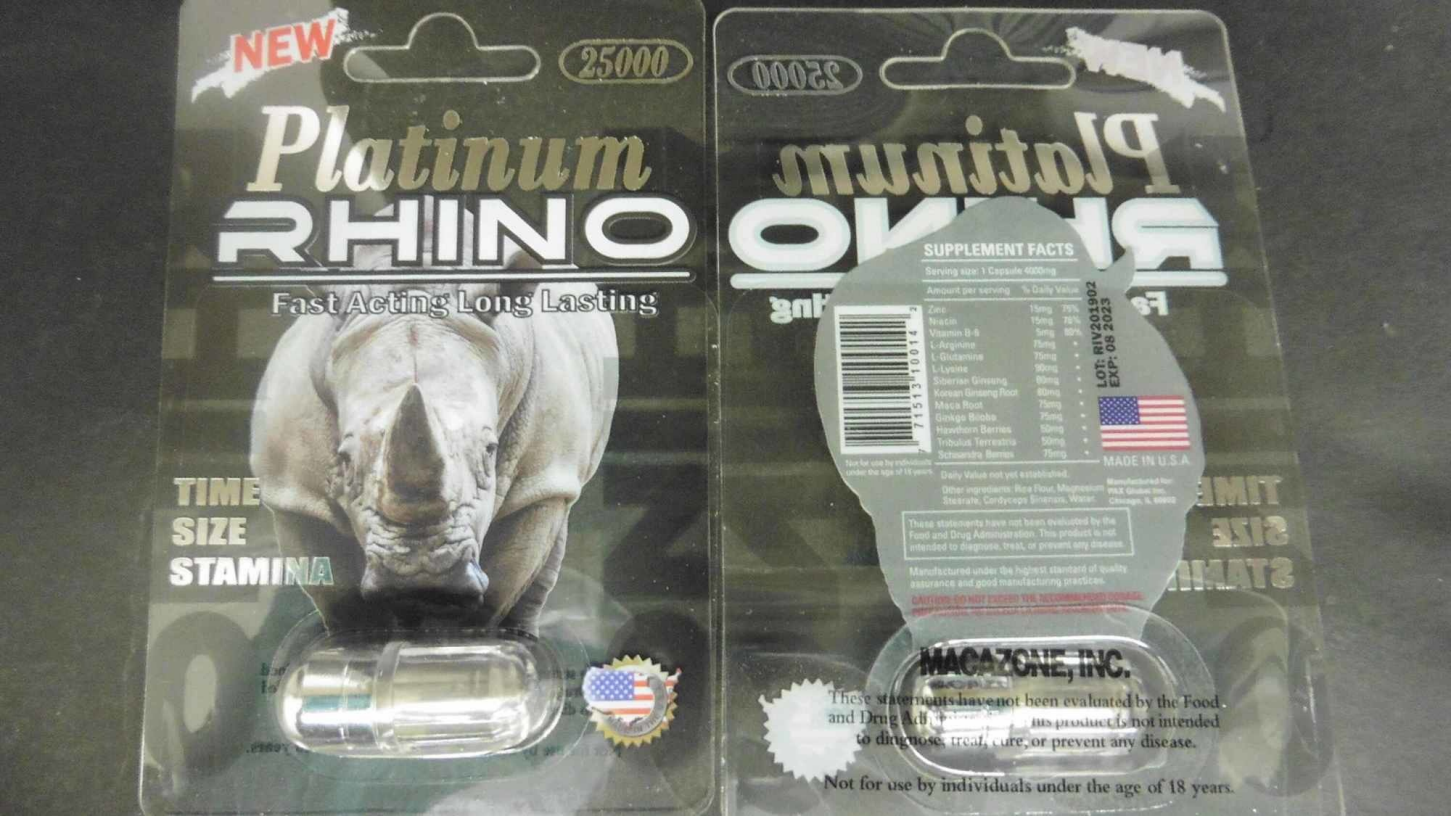
Rhino pills Image courtesy: FDA website (https://www.fda.gov/drugs/medication-health-fraud/public-notification-platinum-rhino-25000-contains-hidden-drug-ingredient).

ED has a temporal relationship to poor male vitality and early CAD, while PDE5i seems to have the highest rates of compliance and satisfaction [6]. Familiarity with sildenafil has revealed that tolerance develops, and non-responders often resort to higher dosages or illegal enhancers to achieve satisfying results [7]. Pricing constraints are an essential factor with prescription PDE5i costs rising due to active patents. The robust market for illegal sexual enhancers is attributed to profit margins, often 1000% more than with other street drugs, while penalties are usually less harsh [4]. There are numerous reports of sildenafil causing acute MI in nitrate-free patients, and despite the low incidence of MI in these patients from PDE5i, these drugs are known to display adverse cardiac events [8,9]. We aim to parse out potential mechanisms based on the premise of a 100% coronary occlusion seen on angiography. PDE5i are known to lower blood pressure on an average of 8/6 mm Hg; this could have been potentiated in our patient who ingested three 950 mg rhino pills, that are known to be 14-200 times over prescription limits each, inducing a steep drop in blood pressure or redistribution of blood via coronary steal through its vasodilatory properties [9]. Albeit vitals checked by emergency services on arrival and repeated in the emergency department were normal with the patient endorsing to never using nitrates. A reasonable rationale suggested by other authors from similar cases is the possibility of sildenafil contributing to acute thrombosis in the absence of a cardiac history by inducing platelet aggregation based on in-vitro studies and canine models [10]. Based on these concerns, most of these authors suggest a cardiac screening in patients considering PDE5i therapy. Additionally, studies describe the relationship between the occurrence of an MI and sexual intercourse, with the risk being the highest within the first few hours after completion. In the presentation paralleled by our patient, he began experiencing symptoms shortly after completing intercourse, which was considered the trigger factor in reports indicting sildenafil to MI. The calculated Naranjo score that estimates the probability of adverse drug reactions using aspects of this case is 4, ruling out the possibility that the STEMI was not due to rhino. Lastly, rhino and other sexual enhancers are often blended with impure substances, such as talcum powder, inks, and paint with the possibility of numerous adverse effects (Figure 6) [3].

## Conclusions

ED is an indicator of early CAD with PDE5i, where users experience an initial positive response with the majority eventually discontinuing or switching therapy. A substantial market for sexual enhancers exists across the US to augment this. A new product named rhino contains up to 200 times the standard enhancement dose, which primarily comprises of sildenafil or tadalafil. These high doses may play a role in acute thrombosis and lead to adverse cardiac outcomes based on canine and in-vitro models, although further studies are required to parse out more information.
